# Multiple cerebral microinfarcts: an uncommon presentation of Cerebral Amyloid Angiopathy-related inflammation

**DOI:** 10.1186/s42466-023-00253-9

**Published:** 2023-06-22

**Authors:** Aikaterini Theodorou, Athanasios Tsibonakis, Ioannis S Pateras, Georgia Kaloudi, Eleni Bakola, Maria Chondrogianni, Elissavet Andreadou, Ioannis G Panayiotides, Georgios Tsivgoulis

**Affiliations:** 1 Second Department of Neurology, School of Medicine, “Attikon” University Hospital, National and Kapodistrian University of Athens, Athens, Greece; 2 Second Department of Pathology, School of Medicine, “Attikon” University Hospital, National and Kapodistrian University of Athens, Athens, Greece; 3 First Department of Neurology, School of Medicine, “Eginition” University Hospital, National and Kapodistiran University of Athens, Athens, Greece; 4 Department of Neurology, University of Tennessee Health Science Center, Memphis, TN USA

**Keywords:** CAA-ri, Cerebral microinfarcts, MRI, Brain biopsy, Case Report

## Abstract

**Background:**

Cerebral Amyloid Angiopathy-related inflammation (CAA-ri) is a distinct but rare subset of CAA. The greater availability of high resolution Magnetic Resonance Imaging (MRI) has currently allowed the increasing recognition and diagnosis of this entity, without the risk of a brain biopsy. However, in rare cases with typical clinical characteristics but uncommon neuroimaging findings at presentation, the brain-biopsy is required for an early and reliable diagnosis.

**Case description:**

A 71-year-old man with arterial hypertension presented due to 1-week history of headache, vomiting, disorientation and impaired consciousness. Brain MRI revealed multiple acute cortical/subcortical microinfarcts, scarce microbleeds, extensive right parietooccipital and left frontotemporal leptomeningeal enhancement. After an extensive diagnostic work-up, excluding infectious, neoplastic and autoimmune etiologies, the patient underwent brain-biopsy. Histology disclosed amyloid deposition in an arteriolar wall and the patient fulfilled diagnostic criteria for probable CAA-ri with supporting pathology. He received intravenous methylprednisolone, followed by oral tapering with steroids showing clinical and radiological improvement with complete resolution of gadolinium enhancement. Follow-up MRI revealed an increase of cerebral microbleeds and the patient fulfilled CAA-ri neuroimaging criteria.

**Conclusions:**

This case highlights the importance of continuous vigilance from clinical neurologists to detect CAA-ri diagnosis and the diagnostic value of brain-biopsy in CAA-ri patients with atypical neuroimaging presentation, such as acute microinfarcts. The early diagnosis and the prompt treatment initiation can improve the prognosis and the evolution of this rare disorder.

## Introduction

Cerebral Amyloid Angiopathy-related inflammation (CAA-ri) is a distinct subset of Cerebral Amyloid Angiopathy (CAA). It is a small vessel disease, characterized pathologically by amyloid-Aβ deposition in the media/adventitia of cortical/leptomeningeal arterioles with perivascular nondestructive inflammation [1]. CAA-ri is a disease of the elderly with a mean age of 70 at diagnosis, without obvious gender predominance [1, 2]. The most common clinical manifestations include cognitive decline, focal neurological deficits, encephalopathy, seizures and headache, whereas typical neuroimaging findings are hyperintense T2/Fluid Attenuated Inversion Recovery (FLAIR) white matter lesions complicated with lobar microbleeds, leptomeningeal or intraparenchymal gadolinium enhancement in the areas of T2/FLAIR hyperintensities and focal/disseminated cortical superficial siderosis [1, 2]. Radiological findings, including lobar hemorrhage and ischemic infarcts, especially microinfarcts represent very rare neuroimaging manifestations [3].

## Case report

**Fig. 1:**
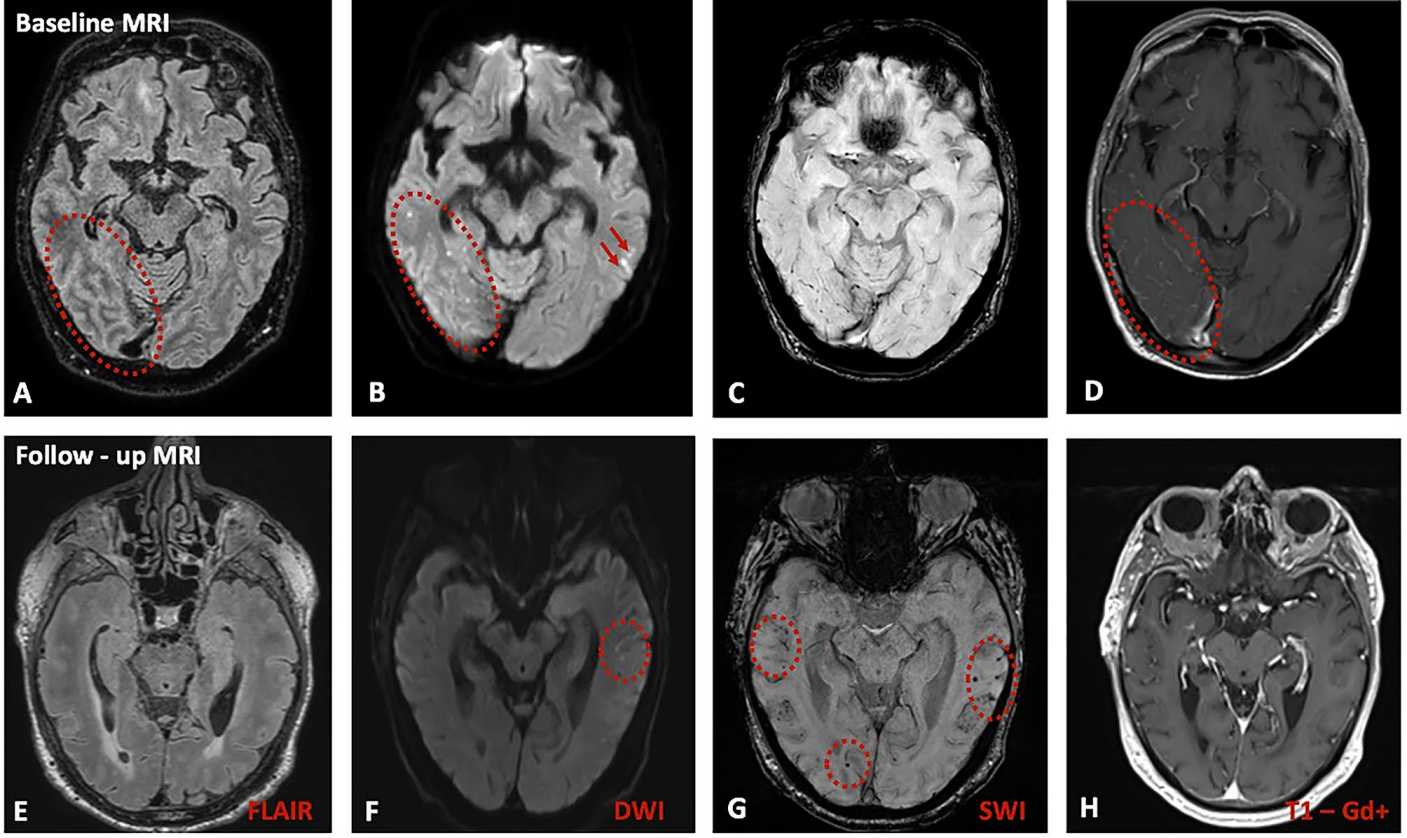
MRI findings at initial presentation and after corticosteroid treatment. Baseline-MRI showed FLAIR subarachnoid hyperintensities (Panel-A; red dotted circle), multiple cortical/subcortical microinfarcts in Diffusion-Weighted-Imaging (Panel-B; red dotted circle and red arrows), scarce microbleeds in Susceptibility-Weighted-Imaging (Panel-C) and leptomeningeal gadolinium-enhancement right parietooccipital (Panel-D; red dotted circle). Follow-up Imaging two months post-corticosteroid initiation disclosed resolution of FLAIR-hyperintense lesions (Panel-E), new subcortical microinfarcts (Panel-F; red dotted circle), increase of cerebral microbleeds (Panel-G; red dotted circles) and complete resolution of gadolinium-enhancement (Panel-H)

A 71-year-old, functionally independent man, with known arterial hypertension presented due to 1-week history of headache, vomiting, disorientation, apathy, visual hallucinations and impaired consciousness. The patient underwent brain Magnetic Resonance Imaging (MRI), revealing multiple acute cortical/subcortical microinfarcts accompanied by scarce cortical/subcortical microbleeds. T1 post-gadolinium sequences showed extensive right parietooccipital and left frontotemporal leptomeningeal enhancement, eventually suggesting an underlying primary central nervous system angiitis (PACNS) (Fig. 1, Panels A-D).

**Fig. 2:**
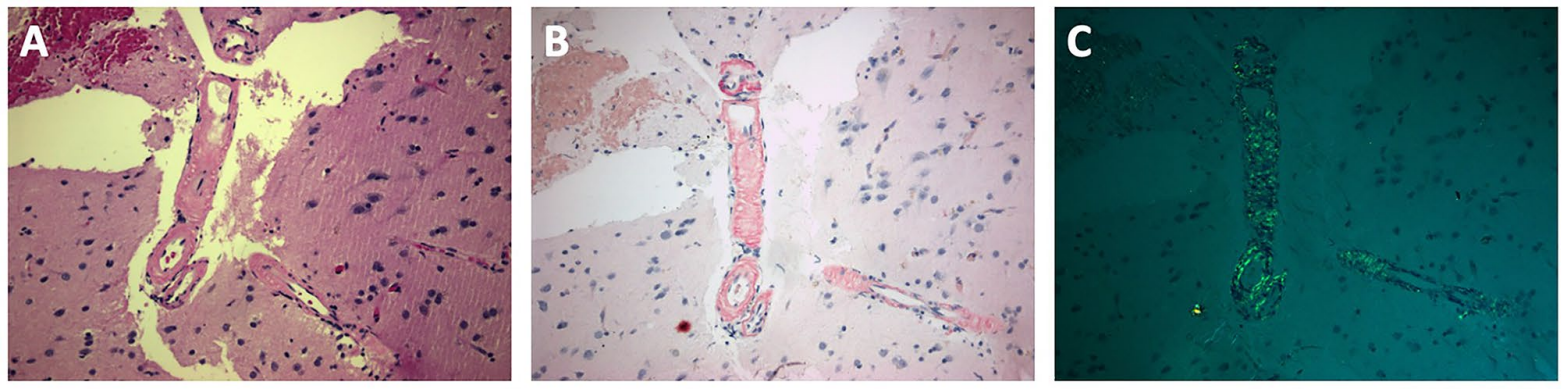
Brain Biopsy. Cerebral parenchyma containing an arteriole with eosinophilic amorphous deposit in its wall; haemorrhage in upper left area (Panel-A: Haematoxylin and Eosin stain, x20). The arteriolar wall stains red with Congo Red stain (Panel-B: Congo Red stain, x20) and displays apple-green birefringence under polarized light (Panel-C: Congo Red stain, x20)

Lumbar puncture was performed, showing increased cerebrospinal fluid (CSF) white blood cells (50/µL; normal value:≤5/µL) and total protein (238 mg/dl; normal value:15–45 mg/dl) with normal CSF-glucose/Serum-glucose ratio (0.62; normal value > 0.6) and normal oligoclonal bands (IgG-Index: 0.8; normal value:<0.6). An extensive diagnostic work-up excluded infectious, neoplastic and autoimmune etiologies and the digital subtraction angiography did not disclose any angiographic abnormalities, such as irregular notched appearance of cerebral arteries, suggestive of PACNS. Before treatment initiation, the patient underwent brain-biopsy to confirm our diagnostic suspicion, however histology disclosed amyloid deposition in arteriolar wall (Fig. 2, Panels A-C). The patient fulfilled diagnostic criteria for probable CAA-ri with supporting pathology [2]. The Apolipoprotein-E (ApoE) genotype was ε3/ε3. He was treated conservatively with intravenous methylprednisolone, followed by oral tapering with prednisolone, showing gradually clinical improvement; one month after corticosteroid initiation he was fully-oriented without any psychiatric signs or focal neurological deficits and follow-up brain MRI revealed complete gadolinium enhancement resolution, indicative of radiological improvement. However impressive increase of cerebral microbleeds was documented (Fig. 1, Panels E-H). Typical T2/FLAIR hyperintense white matter lesions were absent in initial and follow-up MRI images.

## Discussion

CAA-ri has been increasingly recognized due to the more comprehensive understanding of this cerebrovascular disease by vascular neurologists. The greater availability of high resolution MRI has allowed reliable diagnosis of probable/possible CAA-ri according to recent diagnostic criteria, avoiding brain-biopsy risks [4]. Susceptibility Weighted Imaging (SWI) at presentation is undoubtedly more sensitive in detecting earlier more hemorrhagic lesions compared to previously used sequences (Gradient-Echo T2* Weighted Imaging) [5]. Consequently, brain-biopsy is nowadays underutilized due to periprocedural risks/complications, that are relatively uncommon 0.5-1%.

However, patients with characteristic clinical manifestations, including cognitive decline, focal deficits and encephalopathy but atypical neuroimaging findings, such as microinfarcts or lobar hemorrhages, need brain-biopsy to be finally diagnosed with CAA-ri [3]. Biopsy-proven CAA-ri diagnosis in patients with uncommon radiological presentation, including multiple acute cortical/subcortical microinfarcts and normal SWI or alternatively rare microbleeds, indicates probably an earlier disease stage. Follow-up MRIs disclose in the majority of these patients a wider spectrum of typical radiological manifestations, including T2/FLAIR hyperintense white matter lesions, microbleeds and gadolinium enhancing lesions at a later disease stage.

The clinical suspicion of CAA-ri, which leads rarely and in diagnostic challenging cases to brain-biopsy conduction is of highly importance [2]. The differential diagnosis includes infectious diseases where high doses of corticosteroids are contraindicated, or malignancies such as central nervous system lymphoma, where early initiation of corticosteroids could influence lesion’s dimensions or histopathological findings. CSF findings, including elevated protein levels and cell count with negative oligoclonal bands are often described among patients with CAA-ri, without however high specificity. APOE gene testing is performed, since the APOE ε4-allele has been characterized as the only genetic risk factor for CAA-ri. Furthermore, the early diagnosis before treatment initiation is of paramount clinical importance, since treating physicians are able to upgrade earlier the initial therapy with glucocorticoids (intravenous methylprednisolone, 1000 mg per day for 5–7 days, followed by 1 mg/kg oral prednisone daily with slow tapering off over months) to immunosuppressants (including cyclophosphamide, mycophenolate mofetil, azathioprine, IVIG, or rituximab as add-on therapies) in refractory cases [1, 2].

In conclusion, the continuous vigilance to detect new probable/possible CAA-ri cases is essential for the overall outcome of these patients. Moreover this case highlights the diagnostic value of brain-biopsy in CAA-ri patients with atypical neuroimaging presentation, such as acute microinfarcts. Earlier diagnosis and prompt treatment initiation with glucocorticoids or even immunosuppressants may lead to better prognosis and improve the natural disease course.

## Data Availability

The data that support the findings of this study are available in the text.
